# Unlocking the Potential of Teff for Sustainable, Gluten-Free Diets and Unravelling Its Production Challenges to Address Global Food and Nutrition Security: A Review

**DOI:** 10.3390/foods13213394

**Published:** 2024-10-25

**Authors:** Mary Adepoju, Carol Verheecke-Vaessen, Laxmi Ravikumar Pillai, Heidi Phillips, Carla Cervini

**Affiliations:** Magan Centre of Applied Mycology, Cranfield University, Cranfield MK43 0AL, UK

**Keywords:** teff, gluten free, sustainable diet

## Abstract

Sustainable diets, as defined by the Food and Agriculture Organisation, aim to be nutritionally adequate, safe, and healthy, while optimising natural and human resources. Teff *(Eragrostis tef)*, a gluten-free grain primarily grown in Ethiopia, has emerged as a key contender in this context. Widely regarded as a “supergrain”, teff offers an outstanding nutrition profile, making it an excellent choice for people with gluten-related disorders. Rich with protein, essential amino acids, polyunsaturated fats, and fibre, and abundant in minerals like calcium and iron, teff rivals other popular grains like quinoa and durum wheat in promoting human health. Beyond its nutritional benefits, teff is a hardy crop that thrives in diverse climates, tolerating both drought and waterlogged conditions. Due to its resilience and rich nutrient content, teff holds the potential to address nine of the 17 United Nations’ Sustainable Development Goals (SDGs), including SDG 1 (no poverty), SDG 2 (zero hunger), and SDG 3 (good health and wellbeing), which are tied to improving food and nutrition security. However, teff production in Ethiopia faces significant issues. Traditional farming practices, insufficient storage infrastructure, and food safety challenges, including adulteration, hinder teff’s full potential. This review explores teff’s dual role as a nutritious, sustainable food source and outlines the key challenges in its production to conclude on what needs to be done for its adoption as a golden crop to address global food and nutrition security.

## 1. Introduction

The need for alternative food options is increasing with the rise of gluten-related disorders (1.4%) and health-conscious consumers [1]. Such alternative solutions should be sustainable, be resilient to climate change, and promote biodiversity while being compatible with the gluten-free (GF) market’s needs.

The GF market represented an economic value of USD 5.9 billion in 2021 with a predicted annual growth rate of 9.8% [2]. Despite the growth of the GF market, living with gluten allergy remains expensive [3]. Many convenience foods, breakfast cereals, and beverages are made primarily from wheat and barley, rye, and oats [4]. This challenge reduces the dietary options of patients, and threatens food security, especially in developing countries.

Several studies have been conducted to investigate the economic impact of living with a gluten-related disorder. For example, a study carried out in the USA [5] reported an inconsistent availability of GF products across premium and low-cost stores, with the overall cost of these products being 183% higher than that of their gluten-containing counterparts. In a survey carried out in Poland, [6] reported that some adult subjects could not observe strict adherence to gluten-free diets due to the higher cost of GF foods, limited availability, and a smaller selection of GF products when compared to conventional food products. A study carried out in Mexico by [7] reported a less than 10% availability rate of gluten-free products out of the 16 supermarkets and 10 health food stores investigated. The cost ratio for these products was reported to be seven to ten times higher than that of their gluten-containing counterparts. Studies reported by [8] for Moldova indicate that adherence to a GF diet among patients with gluten-related disorders varies from 44 to 90%. Several studies across the UK [9,10,11,12] report the availability of convenient GF foods, such as breads, pasta, breakfast cereals, biscuits, cakes, etc., and their cost. These reports show the availability of GF foods but mostly in premium markets and that they are significantly 400% more expensive than their gluten-containing counterparts. The quality of GF products and their price are conclusively described as major issues globally. Most of these reports conclude by describing this overall situation as one which will continue to negatively impact poor socioeconomic groups’ ability to afford as well as adherence to GF diets. The inability to strictly adhere to GF diets will consequently increase morbidity and healthcare costs.

Moreover, rapid climate change coupled with loss of biodiversity and reduced water availability are key drivers towards the adoption of new climate-resilient crops (CRCs) capable of withstanding environmental stress [13,14]. CRCs are intended to maintain or enhance crop yields in challenging climate-related conditions (e.g., drought stress, higher average temperature) [15]. Despite such clear advantages, the adoption of CRCs among smallholder farmers has been slower than expected in certain cropping systems.

Teff (*Eragrostis tef)* is a nutrient-dense GF cereal grass that is grown and consumed in Ethiopia. The country produced 4.4 million tons of teff in 2020, on approximately 2.7 million hectares with an average yield of 1.6 metric tons per hectare [16], being the world’s largest producer of teff, contributing to approximately 85% of the global production [16]. Teff production in Ethiopia has gone up by 7.4% each year since 2010, mainly due to an expansion of cultivated farmland rather than improved technologies. The Oromia and Amhara regions are the largest teff-producing areas, accounting for about 87.8% of the national teff production volume [17]. Teff grows well in a wide range of agroclimatic conditions, being cultivated in mid-altitude and highlands regions (1500–3000 m), where annual rainfall is on average between 500 and 1200 mm, with temperatures ranging from 15 to 25 °C, and in vertisols and heavy clay soils with pH values from 6.2 to 8.5 [18,19,20,21,22,23]. In Ethiopia, teff is produced by small-scale farmers, who grow one hectare per household and account for more than 90% of the overall average farm holding [24]. It is a major staple crop indispensable to the livelihoods of many Ethiopians. It is used in the making of the Ethiopian traditional sourdough flat bread [25], called *injera,* and it has been used in the production of GF alternatives, such as pasta, composite bread, beverages [26], infant formula [27], composite complementary infant foods [28], and several other value-added products either as composites or pure teff products [29]. Moreover, teff’s by-products have useful applications in animal feed and construction works, confirming its sustainability and adaptability to the circular economy [30]. In recent years, its cultivation has spread to other parts of the world such as Australia, Cameroon, Canada, China, India, Kenya, the Netherlands, North America, South Africa, Sudan, the UK, and Uganda [28]. A recent study by [31] has shown the potential of teff to be cultivated under Mediterranean climatic conditions. 

Teff can be considered a CRC due to its ability to adapt to a wide range of environments, and its natural resistance to insect pests [32]. Teff seeds remain viable for years in the absence of moisture, and it is resistant to attacks by weevils and other storage pests, making it capable of being safely stored under traditional storage conditions with no chemical protection [33,34]. 

Though teff is still very much underutilised outside its country of origin, it is gradually gaining global prominence [35,36]. It was predicted in 2015 that teff will emerge as a new super-crop with growing demand in the global market [37], and *injera* was predicted to be the next super-food worldwide [17]. The commercial market demand for teff is predicted to increase by 304% by 2030 [38]. The prediction is swiftly becoming a reality because of teff’s unique nutritional and agroclimatic attributes. With the call for a second Green Revolution, teff should be considered as a global staple crop along with the first Green Revolution’s major staple crops we know today [39]. In response to the rising demand for alternative food options and the escalating global climate crisis, this review evaluates teff’s potential as a sustainable crop to ensure food and nutrition security worldwide. The specific objectives included (i) analysing teff’s nutritional properties and health benefits in comparison to more popular cereals, (ii) examining its sustainability in relation to the Sustainable Development Goals, (iii) assessing the current production chain, (iv) identifying food safety challenges with a focus on mycotoxins, and (v) evaluating opportunities in the international market.

## 2. Methodology

A systematic review was carried out using the Google Scholar and Science Direct searching engines. A total of 2243 papers, of which 875 were retrieved from Science Direct and 1368 from Google Scholar, were pooled on 6 September 2024. Sixteen search strings applicable to each of the objectives were used for the search: “teff”, “teff gluten free”, “teff quinoa”, “teff durum wheat”, teff nutrients”, “teff nutrient”, “teff sustainable”, “teff sustainable development goals”, “teff production”, “teff production Ethiopia”, “teff challenges”, “teff food safety”, and “teff mycotoxins”. These were searched within the title, abstract, and keywords of the papers, from 2000 to 2024. After applying different criteria of inclusion (e.g., papers published in a scientific peer-reviewed journal, papers written in English) or exclusion (e.g., papers that are not accessible, papers duplicated within the search), a total of 116 papers fulfilling the inclusion criteria were considered for this study.

## 3. Teff Is a Supergrain

In recent years, teff has received increased interest due to its valuable nutritional properties and its use in GF food products. However, the nutritional qualities of teff and its benefits are still widely unknown. In this section, the macronutrient and micronutrient composition of teff is presented and discussed in relation to durum wheat (*Triticum turgidum),* its closest processed cereal grain with gluten, and quinoa (*Chenopodium quinoa*), a GF seed commonly referred to as a pseudo-grain owing to its shared similarities to common cereal grains.

### 3.1. Macronutrients

Carbohydrates support metabolic activities within the human body and are a major energy source [30]. Based on the degree of polymerisation, carbohydrates can be classified as sugars and complex carbohydrates (starches and non-starch polysaccharides). Starch is the main carbohydrate in cereal grains, and it is constituted of two glucose homopolymers (amylose and amylopectin) that differ in their chemical structure [30]. Amylose is the linear fraction, with a low polymerisation degree, whereas amylopectin is a highly branched fraction. Variation in the amylose/amylopectin ratio, usually 1:3, has a profound effect on the starch properties, which impacts the technological and nutritional properties [40]. In teff, starch makes up 73% of the grain (Table 1), of which 83% is amylopectin and 25–30% is amylose [41,42]. Durum wheat and teff share very similar starch compositions of about 71 g and similar ratios of amylose/amylopectin. Quinoa, on the other hand, has a lower starch content (58.1–64.2 g), of which 77.5% is made up of amylopectin [43] The carbohydrate composition also determines the glycaemic index (GI), which represents the rate of carbohydrate digestion. The GI of a food depends on endogenous factors in the food matrix, such as susceptibility to α-amylase, protein, and lipid content [30]. Based on the GI, foods can be distinguished into having low (<55), intermediate (>55–70), and high (>70) GI content [44]. Consumption of low-GI foods is recommended, as it has been shown that they may help with weight loss and regulation of blood sugar levels, contributing towards reductions in conditions like type 2 diabetes and heart diseases [44]. Teff is reported to have a GI of 74, which is lower than that of wheat (100) but higher than quinoa (53). Such differences in the GI reflect on the different sizes of the starch granules of these crops. Indeed, teff starch granules are very small (2–6 µm), smaller than those of wheat (20–35 µm) but larger than quinoa’s (0.5–3 µm), suggesting that such granules could be hindered by the enzymatic attack of α-amylase, resulting in a lower GI [30,43].

Dietary fibre (DF) represents the non-starch polysaccharide fraction of complex carbohydrates. It comprises a group of different substances in plant foods which cannot be completely digested [45]. DF includes insoluble dietary fibre (IDF), represented by cellulose, water-insoluble hemicellulose, and lignin, and soluble dietary fibre (SDF), including oligosaccharides and non-cellulosic polysaccharides [46]. A high fibre intake is associated with reduced risk of different conditions/diseases such as constipation, type 2 diabetes, and colorectal cancer [35]. The percentage of IDF in teff is 3% (dry base), which is higher compared to those of quinoa (2–2.2%) and wheat (2%) [42,43,47]. Teff is consumed in the whole-grain form or as flour (bran and germ included), since it is impossible to perform any fractionation during the milling process. This greatly contributes to a higher intake of fibre compared to other GF foods made from refined flour where the outer layer of grain containing most of the fibre is removed. The total DF content of teff (8 g/100 g) is slightly lower than that of other cereals, such as wheat (9.5 g), but it is higher than that of pseudocereals like quinoa (7 g) [48].

Proteins are vital for energy, growth, repair, and maintenance of our bodies, serving as structural units, biochemical catalysts, hormones, enzymes, and initiators of cellular death [49]. The protein content of teff is between 8.7 and 11%, slightly lower than that of wheat (15.47%) and quinoa (16.3%) [42,43]. Teff seed storage protein is composed of glutelins (46.6%) and albumins (39.1%), followed by prolamins (12%) [30]. On the contrary, [50] reported that prolamins are the major storage proteins in teff. Such different findings may be attributed to the different methods of protein extraction between these studies or the varieties of teff studied. Teff has an excellent balance of essential amino acids (EAAs). EAAs are those that cannot be synthesised and must therefore come from the diet. In general, teff surpasses durum wheat for all EAAs while having lower amounts of lysine (1.62-fold reduction), isoleucine (1.19-fold reduction), phenylalanine (1.21-fold reduction), and methionine (1.29-fold reduction) than quinoa (Table 1) [30,32,42]. The overall EAA profile of teff makes it a very well-balanced food. Moreover, teff, like quinoa, has no gluten; therefore, it is a perfect grain to be consumed by people with gluten-related disorders.

Cereals can provide a significant quantity of fatty acids in our diet. Fatty acids, especially polyunsaturated fatty acids (PUFAs), are beneficial to growth, development, and long-term health [30]. PUFAs can be distinguished into omega-3 (ω-3) and omega-6 (ω-6). The main ω-3 is α-linolenic acid (ALA) and the main ω-6 is linoleic acid (LA). Although the human body cannot synthesise either of these, they can be used to synthesise other essential fatty acids [51]. Therefore, there has been increasing interest in making sure they are adequately represented within human diet. A healthy diet contains a balance of ω-3 and ω-6 fatty acids. For instance, ω-3 contribute to reducing the risk of cardiovascular disease, cancer, and inflammatory and autoimmune diseases, and some ω-6 fatty acids tend to promote inflammation [40]. Teff has a fat content of 2–2.38%, similar to durum wheat (2–2.5%) but lower than quinoa (4–7.6%) [32,52]. Although a clear consensus has not been reached on the optimum ratio between LA and ALA fatty acids, there are nutritional recommendations that the ratio of LA:ALA in formula for infants needs to be between 5 and 15 [30,53]. In this regard, despite this ratio being lower in teff (5.3) compared to durum wheat (6.9) and quinoa (10.9), it is still considered very valuable and comparable to those of legumes, such as soybean, which are good sources of fatty acids [30]. 

### 3.2. Micronutrients

Teff is also a valuable crop for its mineral content, which may vary depending on different factors such as climatic conditions and variety [54]. Iron, calcium, and magnesium are the main mineral deficiencies occurring in GF products [55]. Therefore, in this section particular attention will be given to these minerals. Teff is considered a good source of iron, especially the red variety (15.7 mg/100 g), resulting in mineral content approximately two to three times that of wheat (3.7 mg/100 g) and higher than that of quinoa (13.2 mg/100 g) (Table 2) [32,43,56]. However, iron’s availability may be affected by saponins and phytic acid present in the seeds, which can act as anti-nutrient compounds capable of chelating bivalent minerals. The fermentation procedure used to prepare *injera* from teff seeds is reported to destroy phytic acid, contributing to the high iron availability in diets in Ethiopia [32,43,56]. This explains the low frequency of anaemia in the Ethiopian highlands, where teff is a staple crop. With respect to calcium, teff contains an excellent concentration (147 mg/100 g) of this mineral which is higher than that of wheat (39.5 mg/100 g) but similar to quinoa (148 mg/100 g). This is significant for people with coeliac disease due to the well-known prevalence of osteopenia and osteoporosis among patients diagnosed with this disease. Consumption of teff can thus contribute to overcoming such secondary conditions related to gluten-associated disorders. The concentration of magnesium in teff (184 mg/100 g) is higher than that of wheat (103 mg/100 g) but half of that found in quinoa (362 mg/100 g) [32,43,56]. Magnesium is a co-factor in many enzymes regulating diverse biochemical reactions in the body, including protein synthesis and muscle and nerve function. Iron, calcium, and magnesium are found in good quantities in teff for a balanced human diet.

Teff is also a good source of B vitamins, which are important micronutrients that keep the nervous system healthy and help the body to release energy from food. Vitamin B1 (thiamine) content is very similar between teff (0.39 mg/100 g), durum wheat (0.35 mg/100 g), and quinoa (0.38 mg/g). Vitamin B2 (riboflavin) content is reported to be about 0.27 mg/100 g, which is higher than that of durum wheat (0.17 mg/100 g) but lower than quinoa’s content (0.39 mg/100 g). With respect to vitamin B3 (niacin), teff (3.36 mg/100 g) surpasses by 4.8-fold quinoa’s content (0.70 mg/100 g), but it is lower than that of durum wheat (5.5 mg/100 g). Vitamin B6 (pyroxidine) content was found to be very similar between teff (0.48 mg/100 g) and quinoa (0.49 mg/100 g), and higher than that of durum wheat (0.41 mg/100 g), [32,43,56].

**Table 1:** Nutritional composition of teff in comparison with durum wheat and quinoa. Values refer to 100 g of uncooked product.

Component	Teff	Durum Wheat	Quinoa	References
**Starch (%)**	70.6–73	71	58.1–64.2	[30,32,42,43,47,48,54]
**Proteins (%)**	8.7–11	15.4	16.3	[30,32,42,43,47,48,53,54]
**Essential amino acids (g/16 g N)**				[30,32,42,53,54]
Lysine	3.7	2.1	6	
Isoleucine	4.1	3.6	4.9	
Leucine	8.5	7.0	6.6	
Valine	5.4	4.1	4.5	
Phenylalanine	5.7	4.8	6.9	
Hystidine	3.2	2.1	3.2	
Methionine	4.1	1.4	5.3	
Threonine	4.3	2.7	3.7	
Tryptophan	1.3	1.1	0.9	
**Fats (%)**	2–2.4	2–2.5	4–7.6	[30,32,42,43,48,53,54]
**Fatty acids (%)**				[30,32,47,53]
Linoleic acid (LA)	35.8	55	47.3	
α -linolenic (ALA)	7.1	7.9	4.4	
LA:ALA	5.3	6.9	10.9	
**Total dietary fibre (%)**	8	9.5	7	[30,32,48,53]
**Insoluble dietary fibre (%)**	3	2	2–2.2	[30,43,54]
**Energy (kcal)**	345–367	339	313–368	[30,32,42,43,48,54]

**Table 2:** Micronutrient composition of teff in comparison with durum wheat and quinoa. Values refer to 100 g of uncooked product.

Component	Teff	Durum Wheat	Quinoa	References
**Minerals (mg/100 g)**				[32,43,56]
Iron	15.7	3.7	13.2	
Calcium	147	39.5	148	
Magnesium	184	103	362	
**Vitamins (mg/100 g)**				[32,43,56]
Vitamin B1	0.39	0.35	0.38	
Vitamin B2	0.27	0.17	0.39	
Vitamin B3	3.36	5.50	0.70	
Vitamin B6	0.482	0.41	0.49	

## 4. Teff’s Nexus with the Sustainable Development Goals

**Figure 1:**
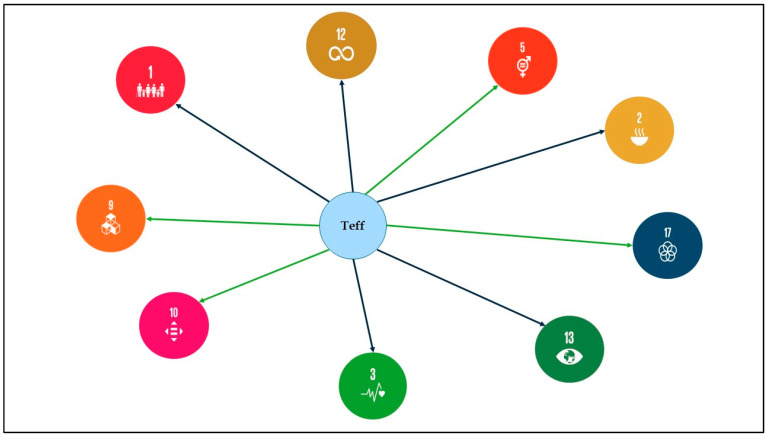
The direct and indirect nexus of teff with nine Sustainable Development Goals (SDGs), emphasising the broader impact of teff on global sustainability. The direct links (blue arrows) include SDG 1 (no poverty), SDG 2 (zero hunger), SDG 3 (good health and wellbeing), SDG 12 (responsible consumption and production), and SDG 13 (climate action); the indirect links (green arrows) include SDG 5 (gender equality), SDG 9 (industry, innovation, and infrastructure), SDG 10 (reduced inequalities), and SDG 17 (partnerships for the goals).

In 2015, the United Nations released its Sustainable Development Goals (SDGs) for 2030 [57]. SDGs 1, 2, and 3 are the three SDGs that are directly linked to food and nutrition security, with the three goals targeted at ending poverty, eliminating hunger, and improving health and wellbeing, respectively. Because sustainability is the nexus of the SDGs, and sustainability is hinged on three pillars, which are environmental viability, economic stability, and social equity, the SDGs in their entirety become a nexus. Teff can contribute directly to the achievement of five SDGs (SDGs 1, 2, 3, 12, and 13) and indirectly to four other (SDGs 5, 9, 10, and 17) of the 17 SDGs defined by the United Nations. Figure 1 shows the nexus of teff with the SDGs.

### 4.1. Direct Links with SDG 1, SDG 2, SDG 3, SDG 12, and SDG 13

In 2021, Ethiopia scored 47.5 out of 100 regarding its food availability within the Global Food Security Index [58]. Further investigation indicated that the prevalence of undernourished Ethiopians decreased by only 0.40% between 2020 and 2021, leading to 21.9% of the country population being undernourished [59,60]. Food and nutrition insecurities are a critical concern throughout Ethiopia, with government officials aiming to address malnutrition via policies, programmes, and large-scale interventions [61]. In Ethiopia, teff is a major staple crop grown by 6.5 million smallholder farmers and it is also an important cash-crop, second to coffee, generating an income of USD 500 million per year for local farmers [36]. In 2015, its most consumed by-product *injera* was estimated to have an export value of approximately USD 10 million [36]. In 2013/2014, the commercial surplus of teff, representing the portion of production sold, was valued at USD 750 million, matching the combined value of all other cereals in the country [16]. Supporting Ethiopian producers and expanding the cultivation of teff out of its places of origin will make it a suitable grain for achieving SDGs 1 and 2, aiming at eradicating extreme poverty and combating hunger. Compared with other cereals, teff is highly tolerable and resilient to extreme conditions, making it a suitable crop for climate action (SDG 13). Teff has shown great ability to grow in a range of agroclimatic conditions. Indeed, it can grow from salty and drought-stressed to waterlogged soils, but it performs better in vertisoils than andosoils with relatively low nitrogen [31]. Moreover, teff requires relatively less water (260–317 mm in a semi-arid environment) than wheat and barley (375 mm) [62,63] and has a short growing season of around 12 weeks [64]. Its phenolic content, which forms a cross-linked cell wall, creates a barrier that makes it resistant to pests or insects, resulting in savings on fertilisers [65,66]. Additionally, just one pound of teff can cover an entire acre in as little as 45 days, whether fertilised or not [67]. These features make it a valuable crop candidate to counteract climate change. Given that *injera* accounts for up to two-thirds of the food consumed in Ethiopia, any effects of global climate change on teff productivity pose significant risks to food security [68]. Recent fluctuations in climatic parameters have become major concerns, negatively impacting teff production and productivity [69,70], which suggests that adaptation strategies will be essential for coping with climate change, and that these may vary based on social and economic factors. Future development initiatives must focus on enhancing perceptions of and scaling up climate adaptation technologies, necessitating collaboration between public and private sectors. Improved policies and investments in extension services should encourage farmer participation in training for effective climate adaptation strategies; ref. [71] found that climate factors like temperature and rainfall may significantly influence net revenue, while [72] predicted an 8.3% increase in teff yields at high altitudes due to climate change, suggesting that higher temperatures may make these areas more suitable for teff cultivation in the short term. Thus, adapting to climate change is critical for Ethiopia’s agricultural strategy and food security and it will require the development of predictive climate models to formulate appropriate adaptive strategies and policies to mitigate the adverse effects of climate change on teff production.

Such valuable agronomic attributes of teff qualify it for adoption as a sustainable crop to address global food and nutrition security while also ensuring sustainable consumption and food production (SDG 12).

Moreover, teff’s outstanding nutritive attributes, discussed in Section 2, qualify it as a valuable crop for ensuring good health and wellbeing (SDG 3). The intake of teff has been shown to improve cardiovascular health, gastrointestinal function, premenstrual symptoms, and immunity due to its abundance of micronutrients and macronutrients [73]. Furthermore, teff is known for its anti-nutrient properties, including phenolic compounds and tannins, which can reduce glucose in the blood, lower the risk of cancer, and help to prevent kidney stones [74]. Nonetheless, these anti-nutrients can inhibit mineral absorption [75]. The processing of teff flour or *injera* can help reduce these anti-nutrient properties, which will increase absorption of nutrients in the body. However, it has been found that during the manufacturing of teff flour, lots of vitamins and minerals are lost [76]; however, for pregnant women, teff reduces the anaemia risk and improves lactation. It also contains vitamins that help in the development of foetuses and infants [77]. Teff grains possess chemo-preventive properties that could repair gene mutations [78]. Despite some limitations, such as bloating, gas, and nutrient absorption inhibition, teff remains a promising grain that can offer a plethora of health benefits to those who consume it [30]. 

### 4.2. Indirect Links with SDG 5 and Beyond 

Teff has also the potential to meet SDG 5’s objective of empowering women to achieve gender equality in a country where rural women represent approximately 70% of the labour force. The authors of [79,80] reported that Ethiopian women play a key role in teff farming activities, from field preparation to harvesting and selling, but they tend to lack education, training, and access to productive agricultural resources. Therefore, gender empowerment and equality should be positioned at the centre of policy makers’ and institutional frameworks to ensure women’s recognition in farming activities. The role of teff in meeting SDGs 1, 2, 3, and 5 would indirectly contribute to the achievement of SDG 10, aimed at reducing inequalities. Improving teff’s value chain through production of food products such as pasta, breakfast cereals, and beverages can contribute to fostering innovation and promoting sustainable industrialisation (SDG 9). The achievement of these SDGs requires concerted efforts by different parties including governments, private sectors, and policy makers to ensure no one is left behind, as stated in SDG 17 (partnership for the goals). 

## 5. Current Teff Production in Ethiopia

**Figure 2:**
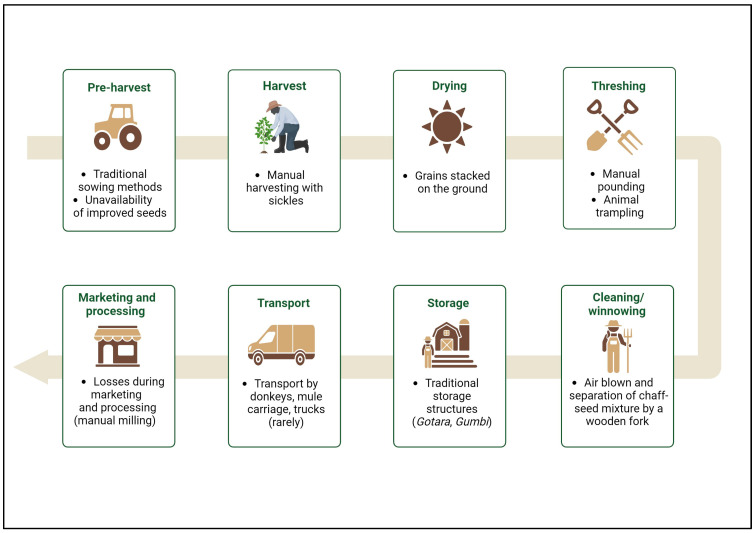
Current stages of teff’s production chain, from pre-harvest to marketing and processing, highlighting key agricultural constraints.

Teff’s production in Ethiopia is constrained by its labour-intensive nature, the lack of mechanisation, and the use of primitive agricultural techniques. The main stages of the teff supply chain and the associated main agricultural constraints currently faced by Ethiopian farmers are highlighted in Figure 2. 

### 5.1. Pre-Harvest 

Teff seed planting starts once the temperature of the soil reaches 18 °C. Early planting might hinder the seed germination, allowing weeds to develop and scavenge the nutrients embedded in the soil [81]. Cultivation of teff is performed by traditional methods (broadcasting and row planting) which have several drawbacks, including high seed requirement and low productivity [17,82]. Teff yields are significantly lower than those of the other most-produced grains in Ethiopia. The total national production of teff in 2022/2023 was 4.4 million metric tons, which was lower than maize (10.2 million metric tons) and wheat (5.7 million metric tons) but similar to sorghum (4.5 million metric tons). The average yield of teff in the same year was 1.6 metric tons per hectare, less than half the yield of maize (4.2 metric tons per hectare) [16,83]. The low productivity is primarily attributed to inadequate crop management practices, poor soil fertility, and inefficient production systems characterised by traditional agricultural practices [84,85]. 

Many research programs have been initiated for breeding different varieties of teff to increase production and yield. As a result of such efforts, in 2012, 35% of teff farmers used enhanced teff seeds compared to 7% ten years before [17]. Even though the availability and affordability of the modified seeds have improved over the past decade, smallholder farmers still find it difficult to use the seeds due to limited access to them and unaffordable prices. White-coloured teff is the most cultivated type as a result of the introduction of the improved variety *Quncho* at a higher premium price [86]. However, white teff only grows in the Ethiopian highlands, it requires the most rigorous growing conditions, and it is the most expensive, representing a status symbol for the wealthiest families in Ethiopia. 

### 5.2. Harvest 

When the teff’s vegetative portions change their colour from green to yellow to indicate maturity, it is harvested. Subject to environmental conditions, this may happen as soon as 45 days following the planting. The period of harvest is essential for quality control because a late harvest might result in grain breaking and colour fading [41].

During harvesting, it is important to take precautions to prevent soil being mixed with the grain. Among small-scale farmers, harvesting is typically performed by hand with sickles, although some larger-scale growers employ harvesting machinery. Due to the weightlessness of individual grain kernels (an average thousand weighs 0.264 g), which makes them easily carried away by the wind, grain loss is typically considerable (25–30%). Ineffective tools and little mechanisation appear to be the main contributing factors to the great loss [5].

### 5.3. Post-Harvest 

The harvested teff stalks are stacked upon the ground or sheets of sack for a short time before being subjected to either pounding with sticks by hand or ox hoof crushing during the threshing process [54]. Threshing, which entails animal trampling, battering, and hammering, is time-consuming and labour-intensive, and significantly results in seed quantity and quality losses. Then, teff grains are separated from the dirt and chaff during the cleaning/winnowing stage by being thrown in the air with a wooden fork [87].

Harvested grains are stored in “Gotera” or “Gota” and “Gumbi”, sometimes referred to as “Togogo”. When stored in “Gotera” within a typical storage environment, teff is suitable for long-term storage for up to 5 years. Research performed by [87] highlighted that “Gumbi/Togogo” is made of specifically developed mud that is joined together with teff straw, while “Gotera” is constructed of bamboo with the interior section lacquered with cattle dung. These facilities have the benefit of being both locally produced and less expensive; however, they are vulnerable to damage from rats, floods, moisture, and fire. Then, teff is transported from the agricultural field to the storage area or the conventional warehouses by animals, usually donkeys. The authors of [87] reported that mule carriages and trucks are also sometimes used. Poor road conditions and a traditional means of transport are also identified as causes of grain losses. Such findings highlight the need for improved agricultural technologies and proper storage facilities to reduce human labour, avoid grain losses, and increase teff productivity.

## 6. Teff Food Safety Challenges

Teff is considered a relatively resistant crop to pest and insect infestation; therefore, the main food safety challenges are represented by chemical hazards. Chemical hazards refer to compounds that have either a low or high molecular weight, which can be produced either naturally or artificially for specific purposes. Based on their physiochemical features and their toxic characteristics, they may negatively impact human health [88]. The main chemical hazards reported in teff include mycotoxins, pesticide residues, and heavy metals. Another issue that has been reported in teff is adulteration, which will also be discussed.

### 6.1. Mycotoxins 

Occurrence of fungal genera such as *Fusarium* and *Aspergillus* has been reported in teff during plantation and storage [89]. These fungi represent food safety concerns, especially as they can produce mycotoxins [90]. Mycotoxins, which are toxic secondary metabolites produced by fungi, undermine both food safety and economy due to non-compliance with international market regulations for export [91]. For example, storage of teff in traditional Ethiopian facilities, characterised by humid conditions, coupled with direct contact with soil, creates a conducive environment for fungal growth and mycotoxin contamination, leading to unavoidable food wastage [92]. Few studies exist on the occurrence of mycotoxins in Ethiopian teff, mainly on aflatoxin B_1_ (AFB_1_) and ochratoxin A (OTA). A summary of the findings is presented in Table 3. The teff samples, meant for human consumption, were collected from threshing yards, and some from traditional storage structures [93]. From the findings of the study, AFB_1_ and OTA were detected in the teff samples. Results of a mycotoxin analysis carried out showed that 22.9% and 27.3% of samples were contaminated with AFB_1_ and OTA, respectively. AFB_1_, produced mainly by *Aspergillus* section *Flavi* species, is a human carcinogen (class 1), while OTA, produced mainly by *Aspergillus* section *Nigri* and some *Penicillium* spp., is a possible human carcinogen (class 2B) [94]. Due to their negative effect on human health, maximum limits exist in Europe and in other countries to regulate their presence in foods. Mean values for AFB_1_ in the tested samples were 3-fold higher than the acceptable limit for EU regulation (2 µg/kg), while those for OTA were found to be 7-fold higher than the EU acceptable limit of 5 µg/kg [91,95]. The high levels of these mycotoxins in teff raise concerns of their fitness for human consumption. In general, contamination by mycotoxins can be effectively reduced to acceptable levels by optimised management integrated along the value chain [82]. Good Agricultural Practices (GAPs), chemical control, biological control, Good Manufacturing Practices (GMPs), and Good Storage Practices (GSPs) are some of the approaches that are useful in preventing/controlling the risk of mycotoxin contamination [96].

### 6.2. Pesticide Residues 

A pesticide is a substance or mixture of substances that is used to prevent, destroy, repel, or mitigate any pest, ranging from insects (insecticides), rodents (rodenticides), and weeds (herbicides) to microorganisms (fungicides, algaecides, bactericides) [97]. Due to their tiny size, teff grains are not typically attacked by insect pests, so pesticides are rarely applied. Additionally, teff is a hardy crop that can tolerate various environmental conditions, including waterlogging, therefore reducing the need for chemical treatments. However, teff can still become contaminated by pesticides from other environmental applications. In this regard, it has been reported by that only 1% of sprayed pesticides reach the target pest, while the remaining 99% pose a threat to human health or to the environment via drift, volatilisation, and leaching [98]. The presence of pesticide residues represents a concern for consumers, as they are known to have harmful effects. The major concern is that they are toxic and can interfere with reproductive systems and foetal development and can cause cancer [99]. Maximum residue levels (MRLs) have been set by the EU to ensure food safety, but not in Ethiopia.

Pesticide use in Ethiopian state farms is estimated at 7.76 kg/ha and less than 0.1 kg/ha in smallholder farms [99]. The pesticide Registration Council of Ethiopia has registered a total of 171 pesticides, of which 159 are currently in use and regulated by the Pesticide Registration and Control Proclamation No. 674/2010 [100]. A study by [101] analysed the presence of pesticide residue in teff grains collected from a local market in the Jimma Zone in Ethiopia. They detected the presence of cypermethrin (0.351 mg/kg), permethrin (0.282 mg/kg), endosulfan (0.014 mg/kg), and DDT (0.296 mg/kg) in teff grains exceeding by 1.16, 5.64, 1.4, and 2.96 times, respectively, the maximum residue limits set by the Codex Alimentarius for grains. In a more recent paper, [99] evaluated the efficacy of household food processing (dough making and baking) in the reduction of pesticide residues in teff. The results suggested that dough making decreased the pesticide residues in the range of 59.9–86.4% and baking in the range of 63.2–90.2% compared to raw teff flour. Such findings highlight the need for strengthening regulations and food safety policies in Ethiopia to limit/avoid the use of certain pesticides (e.g., DDT, which is classified as a possible human carcinogen (class 2B) by the International Agency for Research on Cancer) and to set legislative limits to protect the health of both Ethiopian farmers and consumers. 

### 6.3. Heavy Metals 

Heavy metals are chemical contaminants that are poisonous or toxic to humans. They are naturally found in the soil or can potentially infiltrate the food chain through multiple pathways [102]. The most found heavy metals in teff grains in Ethiopia are cadmium (Cd) and lead (Pb). The contamination of teff with heavy metals is probably due to its small size and suggests increased contact with soil over a larger area, particularly enhanced by traditional agricultural methods of threshing the grain under the hooves of cattle [80]. Cadmium is highly toxic to humans and is a relatively rare element, released to the air, land, and water by human activities. The two major sources of contamination are the production and utilisation of Cd and the disposal of wastes containing Cd [103]. Lead is a chronic or cumulative poison. In humans, Pb can result in a wide range of biological effects depending upon the level and duration of exposure, including increased risk of high blood pressure, cardiovascular problems, and kidney damage [104]. In 1997, the government of Ethiopia established the Environmental Protection Authority (EEPA) for the overall protection of the environment against all physiochemical and heavy metal contaminations. The authors of [105] collected teff grain from the largest teff-producing areas in Ethiopia (Bahir Dar, Debre Markos, and Bure); see Table 4. Lead was detected in the white and red teff samples, with an average concentration of 2.03 mg/kg, which was 10-fold more than the limit established by the European regulations (0.2 mg/kg) [106]. In the literature, teff samples were found on average to exceed by 12-fold the EU regulation limit of 0.1 mg/kg [105,107]. According to [105], heavy contamination may be due to various agricultural activities such as the usage of fertiliser, pesticides, and other industrial products. A way teff can easily be contaminated with heavy metals is through crop rotation and intercropping. Teff is usually subjected to crop rotation with onion, bean, chickpea, and lentil [108]. Onion is a crop that needs many inorganic fertilisers and pesticides for its growth. Therefore, residues of the fertiliser or pesticides used on this soil will be left when teff is planted. Farmers should avoid crop rotating or intercropping with crops that use many fertilisers or crops that are not pest-resistant to avoid the accumulation of heavy metals in teff. Improvements in GAPs and GSPs are also some of the ways to prevent contamination by heavy metals [105].

### 6.4. Adulteration

Teff is susceptible to adulteration within its supply chain. Such adulteration can pose serious health risks to consumers, especially in developing countries like Ethiopia where resources are limited [109,110]. Adulteration has been encouraged by the rise of teff prices in Ethiopia due to the involvement of brokers in its distribution [111]. Several studies have identified common adulterants (e.g., water, sugar, starch) in food in Ethiopia [112,113,114]. With respect to teff, adulterating of teff flour with saw dust (Jesso or “sagatura” in the local language) to make *injera* for sale has previously been reported [115]. Recently, ref. [116] reported that it is a common practice to adulterate teff grains with low-cost materials throughout the supply chain. They identified chaff, soil and sand, and dukka (a combination of non-edible substances separated from teff grains) as the main adulterants, with average contamination levels of 1.17–8.07%, 1.29–7.23%, and 8.93–37.1%, respectively. They also found microbial contaminants (*Escherichia coli*, *Salmonella*, mould, and yeast) and a decrease in the nutritional value of the teff grains due to the adulteration process. These findings underscore the urgent need for collaborative efforts to prevent and control teff adulteration, ultimately ensuring quality and safety for consumers. 

## 7. Teff in the International Market

In 2006, the Ethiopian government imposed a ban on the international market to prevent exportation of unprocessed teff grain and flour [69]. The ban aimed to ensure food security within the country to ensure that the local teff price could be regulated to an affordable level for poor consumers and to discourage smallholder farmers from being driven out of business [21,30]. *Injera* could still be exported and was mainly bought by Ethiopians living in northern Europe, the Middle East, and North America. However, the export ban failed to lower the local teff price due to the increasing demand linked to the continuing population increase, alleged smuggling, and an increase in the volume of exported *injera* [5,30,59,70]. Rather than achieving its intended goal, the ban not only disrupted the trade advantage Ethiopia could have had in the international trade, but also discouraged teff producers and traders from putting in effort to improve its yield, production, market, and value chain [5,17,21]. However, following a 40% increase in yield due to investment into mechanisation and improved farming techniques by the Ethiopian government, the export ban was partially lifted in 2015 [71]. To ensure that the domestic production would not be minimised, the export licences have only been granted to 48 commercial farmers who had not cultivated the plant before. The increasing demand for and general interest in such a crop for its nutritional and agronomic features should encourage the country to speed up the adoption of modern agri-technologies and to boost research. Nowadays, *injera* is exported to various countries including the United States, Sweden, and Norway, with Ethiopian food companies making good profits (e.g., Mama Fresh made approximately USD 1.5 million in 2023) [72]. Because of its potential economic success, other regions, including the USA and Europe, are already cultivating teff and selling it in domestic markets.

## 8. Conclusions

Some of the key challenges with GF foods lie in their availability, cost, and often lower nutritional and sensory quality. Teff is a resilient food crop with promising nutritional properties like wheat, yet is naturally GF. It is rich in fibre and provides macro- and micronutrients, making it highly valuable in addressing nutritional needs.

As a climate-resilient crop, teff offers an excellent opportunity to advance the United Nations SDGs by contributing to sustainable food systems. All these attributes qualify it as a potential crop for a sustainable GF diet, which is crucial in addressing food and nutrition security because of the strong link that has been established between compliance to gluten-free diets and food security in coeliac patients. However, challenges in teff’s production chain, primarily due to its small grain size and limited agricultural technologies, need to be addressed to unlock its full potential. Initiatives such as the second Green Revolution are essential to promote mechanisation and upscaling, which would contribute to reducing post-harvest losses and enhancing overall yields. Additionally, governmental, research, and policy makers’ initiatives (e.g., training for farmers, financial incentives) are needed to encourage farmers to adopt Good Agricultural Practices, as current practices can lead to contamination with dangerous chemical hazards and adulteration that could hinder consumers’ health and challenge accessibility for lucrative international markets such as the EU or UK. A holistic research approach will be vital in supporting these efforts, encouraging the expansion of teff cultivation in other regions, promoting the development of new GF food alternatives, and reinforcing sustainable agricultural practices. This will help ensure a stable supply of this nutritious, gluten-free grain for consumers globally, ultimately contributing to food and nutrition security.

## Figures and Tables

**Table 3:** Occurrence of mycotoxins in teff samples in Ethiopia.

Mycotoxins	Number of Samples	Positive Samples (%)	Mean (μg/kg)	Maximum Limits for EU Regulations (μg/kg)	References
Aflatoxin B_1_	35	22.9	5.1	2.0	[91,93,95]
Ochratoxin A	33	27.3	32.7	5.0	

**Table 4:** Occurrence of heavy metals in teff samples in Ethiopia.

Heavy Metals	Number of Samples	Mean (μg/kg)	Maximum Limits for EU Regulations (μg/kg)	References
Lead	14	2.03	0.2	[105,107]
Cadmium	14	1.22	0.1	

## Data Availability

No new data were created or analysed in this study. Data sharing is not applicable to this article.
